# *Paraburkholderia fungorum* Photoinactivation by Different Wavelengths

**DOI:** 10.3390/life16030493

**Published:** 2026-03-17

**Authors:** Robin Haag, Martin Heßling

**Affiliations:** 1 Institute of Medical Engineering and Mechatronics, Ulm University of Applied Sciences, Albert-Einstein-Allee 55, 89081 Ulm, Germany; martin.hessling@thu.de; 2 Medical Faculty, Ulm University, Meyerhofstraße 28, 89081 Ulm, Germany

**Keywords:** photoinactivation, UV irradiation, blue light, DCFH-DA assay, ROS

## Abstract

*Paraburkholderia fungorum* (*P. fungorum*) is an environmental bacterium with biotechnological applications, yet clinical isolations raise concerns about opportunistic infection risk. Genetically related pathogens exhibit substantial antibiotic resistance, motivating the investigation of alternative control strategies. This paper investigates *P. fungorum* photoinactivation across ultraviolet (222 nm, 254 nm, 313 nm, and 365 nm) and visible (400 nm and 464 nm) wavelengths including ROS (reactive oxygen species) quantification via DCFH-DA fluorescence assay. A two-way ANOVA analysis demonstrated that the wavelength is the dominant determinant of photoinactivation efficacy (F = 100.4, *p* < 0.001) with ROS generation as a more powerful predictor of inactivation than fluence dose alone (F = 60.6, *p* < 0.001) at 365 nm, 400 nm, and 464 nm. Ultraviolet irradiation at 254 nm achieved the highest efficiency (5.4 log reduction at 24 mJ/cm^2^), while 365 nm irradiation demonstrated a high efficacy of 5.2 log reduction at 122 J/cm^2^ with extraordinary ROS production (12,642-fold fluorescence increase). Conversely, inactivation efficiency declined at 400 nm (4.8 log reduction at 378 J/cm^2^ with 122-fold ROS increase) and 464 nm (3.4 log reduction at 3017 J/cm^2^ with lesser ROS detection at 27-fold increase). Wavelength-dependent ROS production correlated directly with bacterial inactivation efficacy, explaining the approximately 500-fold ROS differential between 365 nm and 464 nm. The demonstrated photosensitivity of *P. fungorum* across multiple wavelengths, with the statistical validation of wavelength-dependent mechanisms, provides a foundation for developing practical, mechanism-based phototherapy protocols tailored to specific clinical and environmental decontamination scenarios.

## 1. Introduction

The genus *Burkholderia* comprises Gram-negative, motile, obligate aerobic, rod-shaped bacteria distributed across diverse environments where water and soil are present [1]. Over 120 species have been described, reflecting their versatile occurrence across ecological niches, including soil, water, plant tissues, and animal hosts [2,3]. This metabolic and genetic diversity enables beneficial roles in agriculture and biotechnology, particularly through nitrogen fixation and the degradation of recalcitrant compounds, yet several species are recognized pathogens of plants and humans [1,4,5,6]. In recent years, phylogenomic reclassification has reorganized *Burkholderia* sensu lato into multiple genera, with primarily environmental and plant-beneficial species now designated as *Paraburkholderia*, which is a relatively recent taxonomic separation (established 2014–2018) [7,8,9]. Despite this taxonomic distinction, *Paraburkholderia* species retain homologous antibiotic resistance mechanisms, virulence factors, and biofilm-forming capabilities shared with their *Burkholderia* counterparts; consequently, the literature on *Burkholderia* resistance and inactivation strategies remains directly applicable to *Paraburkholderia* control [10].

Notable pathogenic Burkholderia species include *Burkholderia cepacia* complex (associated with opportunistic cystic fibrosis lung infections; *B. cepacia*), *Burkholderia mallei* (glanders; *B. mallei*), and *Burkholderia pseudomallei* (melioidosis; *B. pseudomallei*) [10,11,12,13]. Most human infections occur as co-infections or in immunocompromised individuals. *B. pseudomallei* infections are particularly significant: an estimated 165,000 annual cases and 89,000 deaths occur globally with melioidosis likely being underreported due to non-specific clinical symptoms [11]. Historically, *B. pseudomallei* was weaponized as a biological agent during World War I, underscoring the pathogenic potential of this genus [14]. As some *Burkholderia* species are beneficial for agriculture, others are known plant pathogens, causing leaf spots, rotten spots, wilts, and vascular diseases across numerous crop species [4,8,15].

Antibiotic resistance is a defining feature of *Burkholderia* species that is driven by multiple mechanisms: (1) outer membrane impermeability due to altered lipopolysaccharide (LPS) structure, (2) RND-family (resistance–nodulation–division) efflux pump systems, (3) inducible beta-lactamases (classes A and C), and (4) biofilm formation [10]. Additionally, two-component systems (TCSs) enable environmental sensing and virulence regulation, facilitating adaptive resistance [12,16]. These overlapping resistance mechanisms substantially reduce antibiotic efficacy and necessitate alternative control strategies.

While taxonomically distinct, the underlying resistance and virulence mechanisms common to both genera may not be fundamentally different. Hence, *P. fungorum*, the bacterium investigated in this paper, shares genomic architecture and genetic organization with clinically important *Burkholderia* species. Specifically, *P. fungorum* encodes a substantial genomic arsenal of virulence determinants including Type III, IV, and VI secretion systems, RND efflux pumps, and homologous adhesion/motility genes (*pilQ*, *hrcS*, *bsaX*), alongside periplasmic stress response genes (*rpoE*, *rseA*, *htrA*) and antibiotic resistance transporters [3]. While classified as an environmental biocontrol agent, the presence of these virulence-associated gene clusters, combined with documented clinical isolations from human septicemia, suggests *P. fungorum* retains opportunistic pathogenic potential comparable to its *Burkholderia* relatives.

Light-based antimicrobial interventions—photoinactivation or photodynamic antimicrobial therapy—evade conventional antibiotic resistance by operating through photophysical and photochemical mechanisms independent of genetic resistance traits. Ultraviolet (UV) radiation, particularly UVC (254 nm), induces direct DNA damage via cyclobutane pyrimidine dimer formation, disrupting DNA replication and transcription process, thereby inhibiting bacterial growth and viability. Visible light (380–780 nm [17]) operates through endogenous or exogenous photosensitizers, which absorb photons, transition to excited electronic states, and generate reactive oxygen species (ROS) including singlet oxygen (^1^O_2_), superoxide radicals ($O_{2}^{\cdot -}$), hydroxyl radicals (OH-), and hydrogen peroxide (H_2_O_2_), leading to membrane damage, protein damage, and bacterial inactivation. The efficacy of visible light inactivation is affected by the presence and concentration of specific endogenous photosensitizers, primarily porphyrins and flavins. Porphyrins, such as coproporphyrin III and protoporphyrin IX, exhibit a strong absorption peak at the Soret band (about 405 nm) and weaker Q bands extending from 500 nm to 700 nm [18]. Flavins, including riboflavin, flavin mononucleotide (FMN), and flavin adenine dinucleotide (FAD), display broad absorption maxima around 450 nm and 370 nm to 375 nm [19,20]. Consequently, irradiation sources near 365 nm can exploit the secondary absorption peaks of flavins as well as the UV tail of the porphyrin Soret band, potentially triggering ROS generation even in the UVA spectrum. The overlap between the emission spectra of the light source and the absorption spectra of these intracellular chromophores is a critical determinant of photoinactivation efficiency.

Several studies have established the photoinactivation efficacy of *Burkholderia* species across different wavelengths, providing a mechanistic precedent for *Paraburkholderia* control. UVC exposure (253.7 nm) at doses of 2.4 mJ/cm^2^ achieved almost 4 log reduction in *B. cepacia* R-1464 [21]. Some members of the *B. cepacia* complex are more durable when exposed to UVC radiation. *B. cepacia* LMG 13010, 12615, 14291, and 16232 have been reduced at a fluence of 7.2 mJ/cm^2^ for 1.5 to 2.5 log reductions [21]. A separate investigation of *B. pseudomallei* exposed to natural UVB filtered sunlight (295–305 nm wavelengths) required 26.5 mJ/cm^2^ per 1 log reduction, including a typical shoulder effect plateau at 70 mJ/cm^2^ [22]. In a photodynamic treatment setting, *B. cepacia* irradiated with blue light (425 nm, 16 mW/cm^2^) combined with curcumin (50 µM) and EDTA (0.4%) achieved 4 log reduction within 30 min (28.8 J/cm^2^ fluence) [23]. Photodynamic antimicrobial chemotherapy (PACT) studies using methylene blue or meso-tetra(N-methyl-4-pyridyl) porphine tosylate with 635 nm red light delivered up to 4-log reductions against *B. cenocepacia*, *B. multivorans*, and *B. cepacia*, though red light alone was ineffective without photosensitizers [24]. These data suggest that *Paraburkholderia* species, sharing homologous photosensitizer targets and cellular machinery, may respond similarly to light-based inactivation.

*P. fungorum* exemplifies the environmental branch of the reclassified genus: isolated from diverse environmental matrices (oil-contaminated soils, plant tissues, and water systems), it demonstrates robust biotechnological potential, including the biodegradation of polycyclic aromatic hydrocarbons (PAHs), phenanthrene, and tolerance to heavy metals and extreme pH conditions [8]. However, rare clinical isolations from human synovial tissue, cerebrospinal fluid, and infectious granulomas have raised concerns about opportunistic pathogenic potential [12,13,25]. Additionally, *P. fungorum* has been reported as a plant-associated bacterium with potential phytopathogenic properties in certain contexts, though its ecological role remains complex and context-dependent. This dual nature environmental utility balanced against emerging infectious disease concerns creates regulatory debate regarding its safe deployment in agricultural biocontrol and bioremediation applications, necessitating effective control strategies independent of antibiotic resistance [4].

Although photoinactivation has been established for pathogenic *B. cepacia* complex and *B. pseudomallei* strains, comprehensive spectral analysis comparing multiple UV and visible wavelengths, combined with mechanistic assessment of ROS production, remains lacking for *P. fungorum*. This limitation is significant given *P. fungorum*’s dual role as an environmental bioremediator and emerging opportunistic pathogen, necessitating the development of light-based control strategies independent of antibiotic resistance mechanisms. This study addresses this limitation by being the first to systematically investigate *P. fungorum* photoinactivation across a broad spectral range. Specifically, we examine six distinct wavelengths (UV: 222 nm, 254 nm, 313 nm, 365 nm; visible: 400 nm, 464 nm) to establish wavelength-dependent inactivation efficacy and correlate bacterial inactivation with ROS production via DCFH-DA quantification. This integrated approach enables us to (1) identify optimal UV and visible wavelengths for *P. fungorum* inactivation, (2) elucidate the role of endogenous photosensitizers in mediating visible light susceptibility through quantitative fluorescence-based ROS detection, and (3) establish *P. fungorum*-specific photoinactivation profiles that can guide the development of targeted phototherapy protocols for environmental and clinical decontamination applications. Specifically, we employed the DCFH-DA fluorescence assay to measure ROS generation across 365 nm, 400 nm, and 464 nm, enabling a direct correlation between ROS production kinetics and bacterial inactivation efficacy. This mechanistic approach—linking fluorescence intensity changes to photoinactivation dose–response curves—provides unprecedented insight into the photochemical pathways governing wavelength-dependent bacterial susceptibility, distinguishing between direct DNA damage (UV) and ROS-mediated mechanisms (visible light). In addition, statistical analyses, including two-way analysis of variance (ANOVA) and Bonferroni-adjusted post hoc *t*-tests, were applied to these datasets to evaluate the main effects and interaction patterns.

## 2. Materials and Methods

### 2.1. Bacterial Strain and Culture Conditions

The bacterial strain used in this study was *Paraburkholderia fungorum* (*P. fungorum*), which is a Gram-negative bacterium obtained from the German Collection of Microorganisms and Cell Cultures (DSMZ, Braunschweig, Germany) under reference number DSM 14061 as a cryo-preserved culture. Culture conditions followed DSMZ recommendations. *P. fungorum* was grown in liquid Medium 535 (M535) to promote planktonic growth and on solidified Medium 535 agar for plate cultures (using 15 g agar per 1 L of M535). The incubation temperature was set to 30 °C.

### 2.2. Sample Preparation

The cryo-preserved culture was spread onto an agar plate using an inoculating loop to promote colony growth at 30 °C. After 48 h, a single colony was picked and inoculated into a cultivation tube containing 3 mL of medium. This pre-culture was incubated overnight in an orbital shaker at 170 rpm. Subsequently, 100 µL of the pre-culture was transferred into 30 mL of Medium 535 in a baffled Erlenmeyer flask to ensure sufficient oxygenation. The main culture was incubated for approximately 12 h at 170 rpm. During this incubation period, the culture reached an optical density at 600 nm (OD_600_) of 0.19 (measured with the spectrophotometer Specord 250 Plus, Analytik Jena, Jena, Germany), corresponding to approximately 10^8^ colony-forming units per milliliter (CFU/mL), representing the stationary phase.

To prepare samples for irradiation experiments, the main culture was washed twice with phosphate-buffered saline (PBS) by centrifugation (Multifuge 3S-R, Kendro Laboratory Products, Langenselbold, Germany) at 4000× *g*. After washing, the bacterial suspension was divided into two 10 mL aliquots: one for the irradiation group and one for the non-irradiated control group.

### 2.3. ROS Detection via DCFH-DA Fluorescence Assay

Dichlorofluorescin diacetate (DCFH-DA; CAS: 4091-99-0; Sigma-Aldrich, Darmstadt, Germany) was prepared as a 10 mM stock solution in dimethyl sulfoxide (DMSO). Before dividing the bacterial suspension into irradiation and control groups, 20 µL of the DCFH-DA stock solution was added to the washed bacterial suspension, achieving a final working concentration of 10 µM. ROS generation was assessed via fluorescence measurements using a microplate reader (CLARIOstar, BMG LABTECH, Ortenberg, Germany). Aliquots of 100 µL from irradiated and non-irradiated control samples were pipetted into a 96-well plate (Greiner 96 F-Bottom, Greiner Bio-One GmbH, Frickenhausen, Germany) in triplicate. Fluorescence was excited at 488 nm, with emission recorded from 511 nm to 600 nm, and total fluorescence intensity was evaluated by calculating the area under the curve (AUC) of the emission spectrum. To validate assay functionality and probe reactivity, positive controls using 10 µL of 3% hydrogen peroxide (H_2_O_2_) were included in each measurement series, confirming robust fluorescence response and excluding probe degradation or depletion [26,27,28].

### 2.4. Light and Radiation Sources

Irradiation was performed using various light sources. For 222 nm irradiation, a UV222™ excimer lamp (UV Medico, Aarhus, Denmark) was used with an irradiance of 1.09 mW/cm^2^. For UV irradiation at 254 nm, 313 nm, and 365 nm, a UV lamp (UVP Handheld UV lamp, Analytik Jena, Jena, Germany) was employed with irradiances of 0.82 mW/cm^2^, 2.34 mW/cm^2^, and 1.69 mW/cm^2^, respectively. Visible violet light irradiation at 400 nm was performed using a narrow-peak black light LED lamp with an irradiance of 35 mW/cm^2^. Additionally, a set of four high-power blue LEDs with a peak wavelength of 464 nm and an irradiance of 279 mW/cm^2^ was used. All spectral and irradiance measurements were performed using a calibrated spectrometer combined with an integrating sphere (CAS 140D, Instrument Systems, Munich, Germany).

### 2.5. Irradiation Setup and Sampling Protocol

For inactivation studies, 10 mL of the bacterial suspension was pipetted into a small Petri dish (55 mm diameter). A custom-designed 3D-printed holder positioned the Petri dish within a cooling device (Thermocell CHB-202, Bioer, Hangzhou, China), which maintained the sample temperature at 8 °C during irradiation. The light sources were positioned directly above the samples. To exclude ambient light, a black 3D-printed tube was placed between the light source (UV Medico lamp, Analytik Jena lamp, or 464 nm LEDs) and the sample. The black light lamp was positioned directly above the setup without a tube.

At defined time intervals, 100 µL samples were withdrawn for both colony counting and fluorescence measurements. Prior to sampling, the bacterial suspension was gently stirred to ensure the homogeneity of treatment. The non-irradiated control was stored in a refrigerator at 8 °C in the dark. Control samples were taken simultaneously with the irradiated samples.

### 2.6. CFU Determination

Bacterial survival was determined by plating samples on agar plates, which were incubated for 48 h to 60 h at 30 °C. Colonies were counted automatically using a colony counter (SphereFlash, IUL S.A., Barcelona, Spain), and data were exported to Microsoft Excel for further statistical analysis.

### 2.7. Fluorescence Spectra Detection and Photosensitizer Assessment

*P. fungorum* cultures were prepared as described in Section 2.2 and subsequently washed. In the final washing step, the PBS volume was reduced to 10 mL (instead of 30 mL) to generate a highly concentrated bacterial suspension suitable for MTP reader fluorescence measurements. A volume of 100 µL of the concentrated *P. fungorum* suspension was transferred into 96-well plates. Samples were excited at 365 nm, 400 nm, and 450 nm to screen for the presence of various photosensitizers. PBS alone served as a blank control for all measurements.

To validate the presence of specific photosensitizers, the following reference compounds were analyzed under identical conditions: riboflavin (0.6 mg) and lumichrome (0.8 mg) were each dissolved in 100 µL PBS and subsequently diluted 1:1000 with PBS. Protoporphyrin IX (PP IX; 1 mg), coproporphyrin III (CP III; 2 mg), and nicotinamide adenine dinucleotide hydride (NADH; 1.3 mg) were dissolved in 100 µL of dimethyl sulfoxide (DMSO) and further diluted 1:1000 with acetone. A final volume of 100 µL of each reference standard was transferred to the 96-well plate for fluorescence quantification.

Excitation wavelengths were selected based on the absorption properties of candidate photosensitizers: 400 nm for riboflavin, lumichrome, PP IX, and CP III; and 365 nm for NADH. Fluorescence emission was recorded across the visible spectrum using the microplate reader (CLARIOstar®, BMG Labtech, Ortenberg, Germany), and spectra were analyzed to identify wavelength-dependent fluorescence signatures characteristic of each photosensitizer.

### 2.8. Data Analysis and Calculations

Calculations, data processing, and visualizations were performed using Microsoft Excel (Microsoft Corporation, Redmond, WA, USA). The fluorescence intensity measured at each time point was corrected for background signals to isolate irradiation-induced ROS generation. Therefore, the contributions of bacterial metabolism-induced fluorescence (DCFH-DA oxidation in non-irradiated bacteria) and sample self-oxidation under irradiation were subtracted from the measured irradiated sample, as presented in Equation (1).

(1) $$\Delta F_{irrad.}(\lambda )=F_{P.\;fungorum+DCFH+irrad.}-F_{P.\;fungorum+DCFH}-F_{DCFH+irrad.}$$

The calculated fluorescence intensity $\Delta F_{\mathrm{irrad}.}$ describes the net fluorescence caused by ROS reacting with DCFH-DA during irradiation. $F_{P.\;\mathrm{fungorum}+\mathrm{DCFH}+\mathrm{irrad}.}$ represents the measured fluorescence of the irradiated *P. fungorum* suspension containing DCFH-DA, $F_{P.\;\mathrm{fungorum}+\mathrm{DCFH}}$ represents the fluorescence caused by bacterial metabolism in the absence of irradiation, and $F_{\mathrm{DCFH}+\mathrm{irrad}.}$ represents the fluorescence of DCFH-DA caused by irradiation in the absence of bacteria. All fluorescence values were first expressed as factors relative to their corresponding controls to describe the relative change rather than absolute fluorescence, allowing an improved comparison between experiments.

For quantification of the possible scavenging effect of DCFH-DA on bacterial inactivation, a protection factor was calculated as the ratio of log reduction obtained without and with DCFH-DA (Equation (2)):

(2) $$\mathrm{protection}\;\mathrm{factor}=\frac{{\log\;\mathrm{reduction}}_{\mathrm{no}\mathrm{DCFH}}}{{\log\;\mathrm{reduction}}_{\mathrm{with}\mathrm{DCFH}}}$$

Values of the protection factors greater than 1 indicate a decreased inactivation in the presence of DCFH-DA, corresponding to a protective or scavenging effect.

In addition, the percentage inhibition of bacterial inactivation by DCFH-DA was calculated to quantify the relative reduction in killing efficiency in the absence or presence of a possible scavenger. This metric is expressed in Equation (3).

(3) $$\mathrm{inhibition}\;[\%]=\frac{{\log\;\mathrm{reduction}}_{\mathrm{no}\mathrm{DCFH}}-{\log\;\mathrm{reduction}}_{\mathrm{with}\mathrm{DCFH}}}{{\log\;\mathrm{reduction}}_{\mathrm{no}\mathrm{DCFH}}}\times 100\%$$

This represents the percentual changes in log reduction between the two conditions (reduction with and without DCFH-DA). The interpretation of this inhibition values is as follows: positive values reflect the protection of bacteria by DCFH-DA acting as a ROS scavenger, values near 0% indicate no relevant scavenging effect, and negative values indicate enhanced bacterial inactivation in the presence of DCFH-DA, suggesting a pro-oxidant or sensitizing effect rather than scavenging.

To contextualize the photoinactivation efficacy of *P. fungorum*, a comparative literature analysis was integrated into the data evaluation. Published inactivation kinetics for phylogenetically related species, such as *B. cepacia* complex, *B. pseudomallei*, and *B. mallei*, were collected to establish a benchmark for UV irradiation and visible light sensitivity. Key parameters, including fluence thresholds and spectral-specific inactivation rates, were extracted and normalized to permit direct comparison with the experimental data obtained in this paper. This comparative framework was used to assess whether *P. fungorum* exhibits distinct durability or susceptibility profiles relative to pathogenic *Burkholderia* strains reported in prior studies.

### 2.9. Statistical Analysis

All statistical analyses were conducted in RStudio (v. 2024.12.0, RStudio PBC, Boston, MA, USA) using R (v. 4.6.0, R Foundation for Statistical Computing, Vienna, Austria) with the following packages: car (v. 3.1.0) for assumption testing and emmeans (v. 1.8.3) for post hoc comparisons.

The dataset included 153 dose-by-wavelength observations from three independent biological trials; technical fluorescence triplicates were not treated as independent observations. Prior to analysis, the normality of quantitative variables was assessed using the Shapiro–Wilk test, which is widely employed in biomedical and biotechnological research for detecting departures from normality. Homogeneity of variance across treatment groups was evaluated using Levene’s test, which is a robust procedure that is insensitive to non-normality and standard in microbiology and photochemistry studies. Although assumption tests indicated deviations, ANOVA was retained because of the substantial effect sizes and its known robustness to moderate violations of normality and homogeneity [29,30,31].

The primary analysis employed two-way factorial ANOVA to examine the independent and interactive effects of fluence and wavelength (categorical: 222, 254, 313, 365, 400, 464 nm) on bacterial log reduction. For statistical analysis, all fluence values were converted to mJ/cm^2^, whereas J/cm^2^ was retained in parts of the text and figures for readability under high-dose visible-light conditions. This approach partitions variance into main effects and their interaction, enabling a direct assessment of whether efficacy depends on wavelength. The statistical model follows (displayed like in R with interaction): log reduction ~ fluence × wavelength + ε (ε: error term of residuals); thus, the fluence effect was interpreted in the context of its interaction with wavelength rather than as a single common dose–response slope across all wavelengths. For visible wavelengths (365, 400, 464 nm), additional models incorporated DCFH-DA treatment status (presence/absence) and, in a mechanistic analysis, measured ROS levels as a covariate.

Following the significant omnibus ANOVA results, pairwise comparisons between wavelength groups were conducted using estimated marginal means with Bonferroni correction for multiple comparisons. The Bonferroni adjustment controls error rate by dividing the significance. This procedure provides a conservative control of false-positive errors across multiple tests. Results are reported with 95% confidence intervals and full *p*-values to enable reproducibility and meta-analysis integration.

## 3. Results

All experiments were performed in biological triplicates (*n* = 3) unless otherwise indicated. Fluorescence measurements were additionally recorded in three technical replicates per biological sample. Data are presented as mean values with their respective standard error of the mean (SEM).

### 3.1. Irradiation Spectra

**Figure 1:**
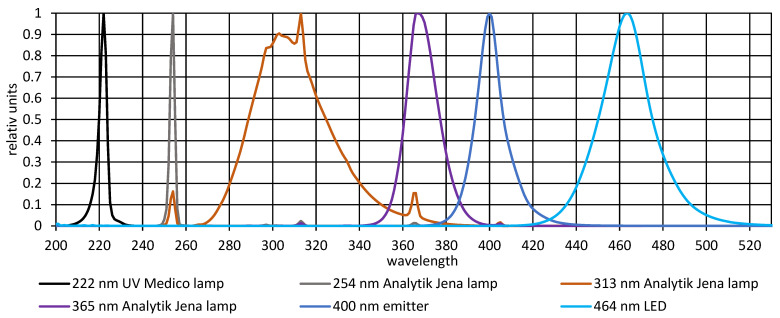
These spectra were emitted from the 222 nm UV Medico lamp, the selectable Analytik Jena lamp including 254 nm, 313 nm, and 365 nm, as well as the 400 nm emitter and 464 nm LED were applied in this paper.

The emission spectra of all six irradiation sources are presented in Figure 1. The 222 nm UV Medico lamp, Analytik Jena lamp (tuned to 254 nm, 313 nm, and 365 nm), 400 nm emitter, and 464 nm LED all demonstrated narrow, well-defined spectral peaks with minimal secondary emission.

### 3.2. Photoinactivation

**Figure 2:**
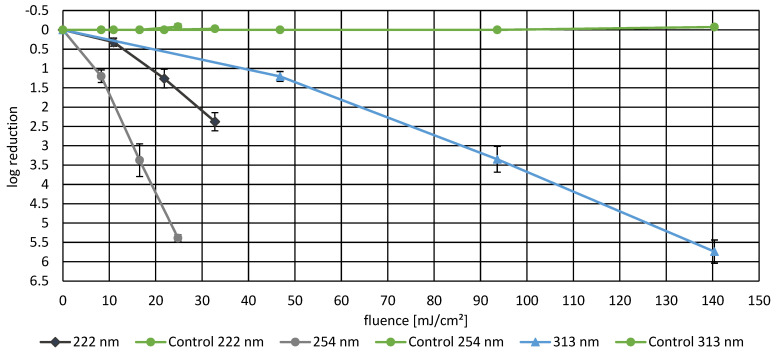
Dose-dependent bacterial log reduction in *P. fungorum* induced by irradiation at 222 nm, 254 nm, and 313 nm. Data points represent mean log reduction values from biological triplicates with SEM. At 222 nm, the log reduction increased from 0.32 ± 0.10 at 10.92 mJ/cm^2^ to 1.26 ± 0.24 at 21.84 mJ/cm^2^, reaching 2.37 ± 0.23 at 32.76 mJ/cm^2^. The 254 nm source was significantly more effective, achieving log reductions of 1.20 ± 0.16 at 8.26 mJ/cm^2^, 3.37 ± 0.42 at 16.53 mJ/cm^2^, and 5.38 ± 0.08 at 24.79 mJ/cm^2^. At 313 nm, the log reduction increased to 1.21 ± 0.13 at 46.8 mJ/cm^2^, 3.35 ± 0.33 at 93.6 mJ/cm^2^, and 5.73 ± 0.30 at 140.4 mJ/cm^2^. Unirradiated controls (shown in green) demonstrated negligible bacterial changes over the experimental duration.

**Figure 3:**
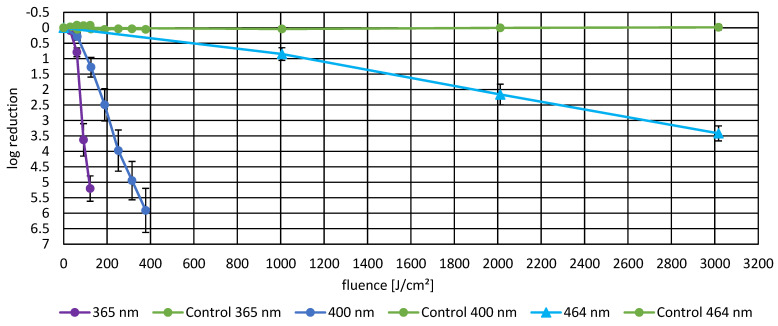
This represents the reduction in *P. fungorum* achieved by irradiation with 365 nm, 400 nm, and 464 nm including their controls. The data points are average values with their respective SEM. Numerical details are displayed in Table 1.

*P. fungorum* was exposed to irradiation at six different wavelengths (222 nm, 254 nm, 313 nm, 365 nm, 400 nm, and 464 nm) to assess the wavelength-dependent effectiveness of bacterial inactivation (presented in Figure 2 and Figure 3). UV irradiation at 254 nm demonstrated the highest inactivation efficiency, achieving a log reduction of 5.38 ± 0.08 at a fluence of 24.79 mJ/cm^2^. In comparison, 222 nm irradiation resulted in significantly lower inactivation with a log reduction of only 2.37 ± 0.23 at a similar fluence level (32.76 mJ/cm^2^), indicating reduced germicidal activity in the far-UVC spectrum. Irradiation at 313 nm and 400 nm both achieved high log reductions of 5.73 ± 0.30 and 5.90 ± 0.71, respectively, at fluences of 140.4 mJ/cm^2^ and 378 J/cm^2^, respectively. Irradiation with 365 nm achieved an inactivation efficiency of 5.20 ± 0.41 log reductions at 122.18 J/cm^2^, and 464 nm required substantially higher fluence to achieve modest inactivation (log reduction of 3.41 ± 0.24 at 3017.3 J/cm^2^), demonstrating that visible wavelengths require significantly more energy for effective bacterial inactivation.

### 3.3. Photoinactivation Including DCFH-DA and Fluorescence Data

**Figure 4:**
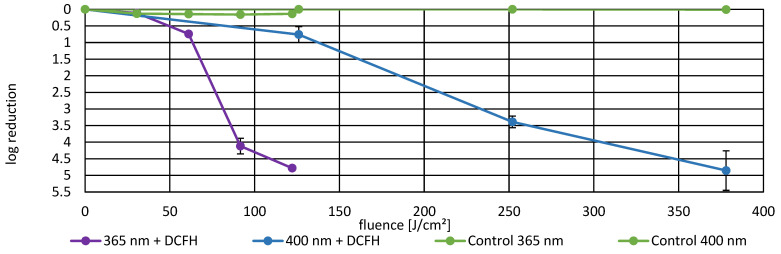
Bacterial log reduction in *P. fungorum* induced by irradiation at 365 nm and 400 nm in the presence of DCFH-DA. Error bars represent standard error of the mean (SEM). Detailed numerical values and additional calculated factors are provided in Table 1.

**Figure 5:**
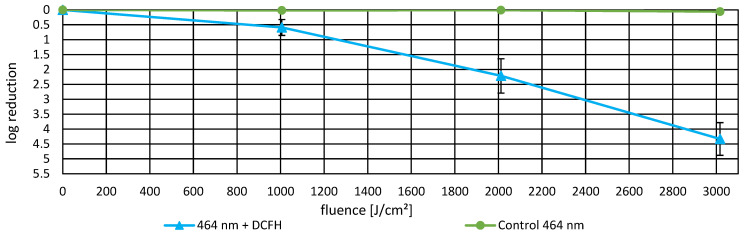
Bacterial log reduction in *P. fungorum* induced by irradiation at 464 nm in the presence of DCFH-DA. Error bars represent the SEM. Detailed numerical values and additional calculated factors are provided in Table 1.

**Figure 6:**
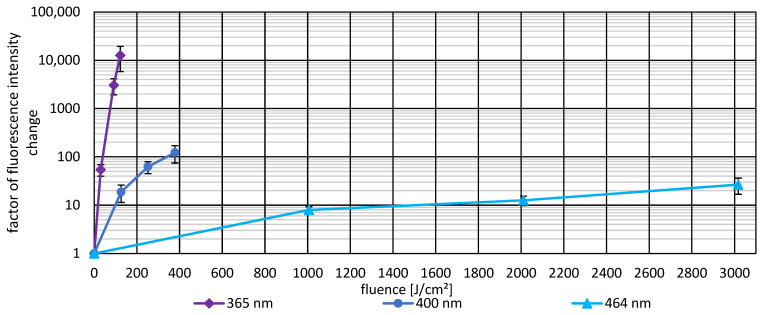
Corrected factor of fluorescence intensity change (ΔF_irrad_.) as a function of applied fluence for *P. fungorum* (in the presence of DCFH-DA) exposed to 365 nm, 400 nm, and 464 nm irradiation. This factor of fluorescence intensity change was calculated as described in Equation (1). The 365 nm irradiation achieved the highest fluorescence intensity (12,642.6 ± 6811.9 fold increase at 122.18 J/cm^2^), followed by 400 nm (122.7 ± 47.9 fold increase at 378 J/cm^2^), while 464 nm showed substantially lower ROS-dependent fluorescence (26.6 ± 9.8 fold increase at 3017 J/cm^2^) despite the highest applied fluence. Errors and error bars represented as SEM.

To investigate whether ROS generation correlates with bacterial inactivation and fluorescence signal and to assess the possible protective effect of DCFH-DA as a ROS scavenger, experiments were conducted at 365 nm, 400 nm, and 464 nm. Bacterial suspensions were irradiated in the presence of DCFH-DA, and log reduction values were compared to calculate protection factors and inhibition as well as the change in fluorescence intensity. Figure 4 presents the dose-dependent inactivation curves for 365 nm and 400 nm. Figure 5 presents the inactivation curve for 464 nm. Table 1 provides detailed quantitative data including protection factors and inhibition percentages for all three wavelengths. Results of the fluorescence induced by ROS generation are presented in Figure 6. Here, ROS generation indicated strong wavelength dependence. At 365 nm and 400 nm, the corrected fluorescence intensity increased exponentially with applied fluence, indicating dose dependent ROS production. In contrast, 464 nm demonstrated substantially lower ROS generation per unit fluence, despite requiring the highest applied fluence to achieve comparable bacterial inactivation. These results indicate that ROS production is wavelength dependent and correlates with the inactivation efficiency observed in the photoinactivation studies.

### 3.4. Fluorescence Spectra Detection and Photosensitizer Assessment

**Figure 7:**
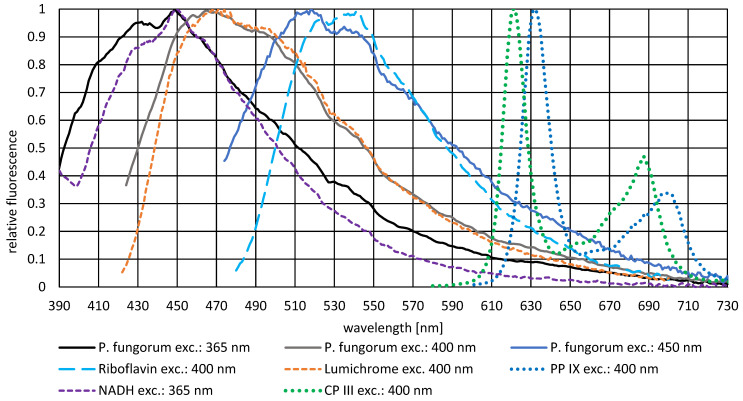
Fluorescence emission spectra of *P. fungorum* and reference photosensitizer compounds. *P. fungorum* suspension was excited at three distinct wavelengths (365 nm, 400 nm, and 450 nm). Reference compounds—riboflavin, lumichrome, NADH, protoporphyrin IX (PP IX), and coproporphyrin III (CP III)—were excited at their respective wavelengths. Spectral overlay reveals close correspondence between *P. fungorum* fluorescence and the flavins (riboflavin, lumichrome) and the pyridine nucleotide (NADH), whereas only porphyrin standards showed detectable emission, but within the *P. fungorum* suspension, no porphyrin like emission was detectable.

Fluorescence emission spectra from *P. fungorum* and reference photosensitizers are presented in Figure 7. When excited at 365 nm, 400 nm, and 450 nm, *P. fungorum* suspension exhibited characteristic fluorescence emission profiles that exhibited substantial spectral overlap with riboflavin, lumichrome, and NADH. Specifically, the peak wavelengths and relative fluorescence intensities of these three compounds closely matched those observed in *P. fungorum*, indicating their likely presence as endogenous photosensitizers within the bacterial cells.

In contrast, neither PP IX nor CP III produced detectable fluorescence signals in the *P. fungorum* suspension under the applied excitation wavelengths. The absence of fluorescence from these porphyrin standards suggests that these molecules are not present in sufficient concentrations within *P. fungorum* cells.

### 3.5. Comparable Literature

To contextualize the photoinactivation efficacy of *P. fungorum*, published UV inactivation data for related *Burkholderia* species were compiled from the literature. Table 2 summarizes the reported log reduction values and corresponding UV fluence doses for various *Burkholderia* strains across different wavelengths, including 254 nm (the germicidal peak), 365 nm (UVA), and 425 nm (visible light with endogenous photosensitizer). These data allow for a direct comparison of photoinactivation sensitivity across the genus and highlight the wavelength-dependent and strain-dependent variation in photoinactivation sensitivity. Inactivation studies including *P. fungorum* remain limited. This compiled literature provides a comparative framework to evaluate related *Burkholderia*/*Paraburkholderia* species and to assess inactivation efficiency. As in this study, it was discovered that *P. fungorum* has a 1 log reduction achievement at about 6 mJ/cm^2^ for 254 nm irradiation. This value is at the upper end of the reported values in the literature. If standard deviation or errors are taken into consideration, the 6 mJ/cm^2^ for 1 log reduction at 254 nm seems plausible. In contrast, irradiation with 365 nm of *P. fungorum* has an estimated log reduction fluence of 70 J/cm^2^, whereas one literature source documented 4.4 mJ/cm^2^ for one log reduction.

### 3.6. Statistical Analysis

#### 3.6.1. Assumption Testing for Parametric Analysis

Prior to conducting the two-way ANOVA, the normality of distributions was assessed using the Shapiro–Wilk test. This revealed significant departures from normality for all quantitative variables: log reduction (W = 0.876, *p* = 5.3 × 10^−10^), fluence (W = 0.54, *p* < 2.2 × 10^−16^), and ROS level (W = 0.173, *p* < 2.2 × 10^−16^). However, given the large sample size (n = 153) and substantial effect sizes observed, analysis proceeded with parametric tests, as an ANOVA is robust against violations of normality under these conditions.

Levene’s test for homogeneity of variance revealed unequal variances across wavelength groups (F_5,147_ = 2.79, *p* = 0.019). This mild heteroscedasticity was acceptable given the large effect sizes observed in the primary analysis and the conservative nature of the overall statistical approach.

#### 3.6.2. Two-Way ANOVA: Effect of Fluence and Wavelength on Log Reduction

A two-way factorial ANOVA was conducted to examine the independent and interactive effects of fluence and wavelength (categorical: 222, 254, 313, 365, 400, 464 nm) on bacterial log reduction (Table 3). The analysis encompassed all six tested wavelengths with n = 147 observations (six measurements with missing values excluded from the original n = 153). This factorial design enables a partitioning of variance into main effects (fluence and wavelength) and their interaction, directly assessing whether photoinactivation efficacy is wavelength-dependent or represents a universal light–dose phenomenon.

The analysis revealed three critical findings concerning the photophysics of *P. fungorum* inactivation (Table 3). First, the wavelength emerges as the dominant efficacy determinant: the wavelength main effect (Sum Sq = 117.2) exceeded the fluence main effect (Sum Sq = 28.2) by ~4-fold, indicating that spectral selection is more powerful than light intensity optimization for enhancing bacterial susceptibility.

Second, the exceptionally large interaction term (F = 100.44, Sum Sq = 416.2, accounting for 78% of explained variance) demonstrates that the dose–response relationship is fundamentally different across wavelengths not merely shifted proportionally. This interaction reveals that *P. fungorum* does not exhibit a unified photoinactivation curve but rather distinct photochemical pathways activated by different spectral regions.

Third, the variance explained by the model (residual sum of squares = 116.9, representing 21% of total variance) indicates that 79% of the photoinactivation variability is accounted for by fluence, wavelength, and their interaction. The remaining 21% may reflect biological heterogeneity (cell-to-cell variations in DNA repair capacity, photosensitizer distribution, membrane composition) and experimental noise (temperature fluctuations, stirring efficiency, photometric calibration).

To validate DCFH-DA as a non-interfering ROS probe, we assessed whether the fluorescent indicator itself affects photoinactivation measurements in Table 4. This validation is critical because antioxidant probes can scavenge ROS or directly protect bacteria from oxidative stress, thereby artificially reducing observed photoinactivation efficacy. A two-way ANOVA was conducted that was restricted to visible wavelengths (365 nm, 400 nm, 464 nm; n = 117) with fluence, wavelength, and DCFH-DA treatment status (presence/absence) as factors (Table 4).

The results demonstrated that the DCFH-DA treatment status exerts no significant main effect on bacterial log reduction (F = 0.374, *p* = 0.542). This finding directly validates DCFH-DA as a non-interfering probe: the bacterial inactivation is equally effective whether or not the fluorescent indicator is present. In contrast, fluence (F = 29.3, *p* < 0.001) and wavelength (F = 50.2, *p* < 0.001) both remain highly significant predictors. Most importantly, the wavelength × fluence interaction persists at exceptional magnitude (F = 155.7, *p* < 0.001, Sum Sq = 302.0), demonstrating that spectral selectivity remains the dominant factor governing (visible) light photoinactivation.

This finding has two important implications. First, mechanistically, the absence of DCFH-DA interference confirms that observed ROS-dependent inactivation is a genuine bacterial vulnerability rather than an artifact of antioxidant depletion by the sample. Second, methodologically, it justifies the simultaneous measurement of ROS (via DCFH-DA fluorescence) and photoinactivation (via CFU count) in the same experimental samples, enabling a direct correlation between ROS production and bacterial inactivation.

To elucidate whether ROS production is the mechanistic driver of wavelength-dependent photoinactivation, we incorporated measured ROS levels as a continuous covariate into a two-way ANOVA model. This analysis was restricted to visible wavelengths (365 nm, 400 nm, 464 nm) where ROS was directly quantified via DCFH-DA fluorescence (n = 35 observations with complete ROS and CFU data). By introducing measured ROS as a predictor alongside fluence and wavelength, we can assess whether the wavelength effect observed in Table 4 is mediated by wavelength-dependent ROS production or remains independent.

The results revealed that the ROS level emerged as a dominant mechanistic predictor, exhibiting the largest mean square value (37.3) and the largest F-statistic (F = 60.6, *p* < 0.001) among all model effects (Table 5). Critically, ROS exceeded fluence in explanatory power (Mean Sq: 37.3 vs. 15.3, corresponding to a 2.4-fold difference), demonstrating that ROS generation is a more powerful driver of photoinactivation than light dose itself. This finding directly addresses the mechanistic question: the visible light inactivation of *P. fungorum* is not simply a function of photon energy delivery; rather, it depends fundamentally on the quantity of reactive oxygen species produced.

The incorporation of measured ROS levels substantially improved model fit, as evidenced by the dramatic reduction in residual variance. The residual sum of squares decreased from 106.6 (Table 4, without ROS covariate) to 17.2 (Table 5, with ROS covariate)—a 6-fold reduction explaining an additional 89.4 units of variance previously attributed to biological heterogeneity or experimental noise. This variance reduction indicates that wavelength-dependent differences in ROS production account for much of the spectral selectivity observed in visible light photoinactivation. In other words, the reason 365 nm is superior to 464 nm is not wavelength itself but rather the wavelength-dependent photosensitizer absorption profiles that leads to ROS generation.

Notably, all main effects and their interaction remained highly significant despite ROS incorporation (fluence: F = 24.8, *p* < 0.001; wavelength: F = 25.5, *p* < 0.001; interaction: F = 38.2, *p* < 0.001), indicating that wavelength and fluence retain independent predictive value beyond their mediation through ROS. This pattern suggests a partially mediated relationship: ROS production is the primary mechanistic intermediate, but wavelength also influences inactivation through wavelength-specific photochemical processes (e.g., differential photosensitizer excitation efficiency, wavelength-dependent cellular uptake of photosensitizers, or direct photon–substrate interactions independent of ROS). The persistence of the wavelength main effect (F = 25.5) even after ROS adjustment underscores the complex, multifactorial nature of visible-light photoinactivation.

Quantitatively, the proportional variance reduction attributable to ROS incorporation can be expressed as follows: ROS accounts for ΔSum Sq = 89.4 of the 106.6 units of residual variance in the non-covariate model, corresponding to η^2^ = 0.839 (83.9% effect size) of the previously unexplained variance. This exceptionally high proportion demonstrates that measured ROS levels explain the vast majority of wavelength-dependent photoinactivation heterogeneity, elevating ROS from a believed mechanism to a quantitatively dominant driver of bacterial susceptibility.

#### 3.6.3. Post Hoc Pairwise Comparisons: Bonferroni-Adjusted Tests

Following the significant two-way ANOVA results, pairwise comparisons between wavelength groups using estimated marginal means (emmeans) with Bonferroni correction to control the familywise error rate across multiple contrasts were performed. Three stratified post hoc analyses addressed distinct experimental subsets: (1) all six wavelengths (222–464 nm; Table 6), (2) visible wavelengths with DCFH-DA treatment status (365–464 nm; Table 7), and (3) visible wavelengths with ROS level covariate (365–464 nm, DCFH-positive samples; Table 8). This hierarchical approach permits both broad spectral comparison and mechanistically refined visible-light analysis.

An analysis of all six wavelengths employed Bonferroni adjustment for 15 possible pairwise contrasts (adjusted α = 0.05/15 = 0.003; Table 6). A clear wavelength hierarchy emerged. The 254 nm wavelength achieved the highest efficacy (emmean = −86,905 ± 11,100), significantly exceeding all other wavelengths (all *p* < 0.0001). The 313 nm wavelength (emmean = −16,204 ± 1970) substantially outperformed all ROS generating wavelengths, demonstrating approximately 1000-fold greater efficacy than 464 nm. Remarkably, the two intermediate UV wavelengths—222 nm (emmean = −28,935 ± 8430) and 313 nm—exhibited statistically equivalent efficacy (*p* = 1.000), indicating that despite their 91 nm spectral separation, both wavelengths engage similar direct DNA-damage mechanisms.

Within the spectrum of 365 nm, 400 nm, and 464 nm, efficacy declined progressively with longer wavelengths. The 365 nm wavelength (emmean = −15 ± 1.1) significantly exceeded both 400 nm (emmean = −5 ± 0.2; difference = −9.68 ± 1.42 units, *p* < 0.0001, representing 3-fold superiority) and 464 nm (emmean = −0.3 ± 0.2; difference = −15.19 ± 1.40 units, *p* < 0.0001, representing 50-fold superiority). Blue light at 400 nm similarly outperformed 464 nm (difference = −5.51 ± 0.48 units, *p* < 0.0001, representing 17-fold superiority). All visible-light comparisons remained far below the stringent Bonferroni-adjusted threshold, providing robust evidence of true wavelength-dependent differences.

A second post hoc analysis examined the visible wavelengths specifically, incorporating DCFH-DA treatment status (presence/absence) as a categorical variable; the results are averaged over treatment levels (Table 7). This analysis employed Bonferroni adjustment for three possible contrasts (adjusted α = 0.05/3 = 0.0167). All three pairwise comparisons remained highly significant. The 365 nm wavelength (emmean = −20 ± 1.7) produced significantly higher log reduction than both 400 nm (emmean = −7.6 ± 0.3; difference = −12.96 ± 1.77 units, *p* < 0.0001) and 464 nm (emmean = −0.4 ± 0.2; difference = −20.17 ± 1.74 units, *p* < 0.0001). Blue light at 400 nm continued to significantly exceed red light at 464 nm (difference = −7.21 ± 0.43 units, *p* < 0.0001). The consistency of these wavelength comparisons across full-spectrum (Table 6) and visible-spectrum-only (Table 7) analyses confirms that visible-light efficacy rankings are robust to analytical subset selection.

The third post hoc analysis examined the mechanistically refined subset of DCFH-positive samples (n = 35) with measured ROS levels incorporated as a continuous covariate (Table 8). Bonferroni adjustment for three contrasts was applied (adjusted α = 0.0167). All three pairwise comparisons remained significant after ROS adjustment but with substantially amplified magnitude differences compared to Table 7. The 365 nm wavelength (emmean = −27 ± 5.1) achieved a markedly higher log reduction than 400 nm (emmean = −7.7 ± 0.7; difference = −19.58 ± 5.28 units, *p* = 0.0027) and 464 nm (emmean = −0.4 ± 0.3; difference = −26.8 ± 5.26 units, *p* < 0.0001). The 400 nm vs. 464 nm comparison remained significant (difference = −7.2 ± 0.7 units, *p* < 0.0001).

A critical observation emerged from comparing Table 7 and Table 8: the 365 nm versus 400 nm difference increased 64% upon ROS adjustment (from (−20) − (−7.6) = −12.4 to (−27) − (−7.7)= −19.3 units), indicating that controlling for measured ROS levels reveals an even stronger wavelength-specific efficacy advantage for 365 nm. This amplification is mechanistically informative: it demonstrates that 365 nm generates disproportionately high ROS relative to 400 nm and that raw efficacy differences (Table 7) underestimate the true wavelength specificity of ROS-dependent photoinactivation mechanisms. In other words, 365 nm’s superiority is driven by its exceptional ROS generation. All Bonferroni-corrected *p*-values remained stringent (*p* ≤ 0.0027), confirming robust statistical evidence for wavelength-dependent ROS production as the mechanistic basis of visible light photoinactivation heterogeneity. There was consistent wavelength hierarchy across all three post hoc analyses. Within the ROS-generating spectrum, efficacy rankings remain constant (365 > 400 > 464), but ROS adjustment amplifies 365 nm versus 400 nm difference by 63%, revealing that wavelength-dependent ROS production is the mechanistic driver. UV wavelengths (254 nm, 313 nm) show orders-of-magnitude superiority via direct DNA-damage mechanisms.

These post hoc analyses establish a robust, three-level wavelength efficacy hierarchy supported by stringent Bonferroni correction: (1) UV domain with direct DNA-damage mechanisms (254 nm >> 313 nm ≈ 222 nm); (2) visible-light domain with ROS-mediated mechanisms (365 nm >> 400 nm >> 464 nm); (3) mechanistic foundation revealed through ROS covariate analysis, demonstrating that wavelength-dependent photosensitizer absorption drives the visible-light hierarchy. These findings provide the quantitative foundation for the Discussion section, where we contextualize these mechanistic insights within broader photochemical and bacteriological frameworks and develop practical implications for wavelength-based decontamination strategies.

## 4. Discussion

The photoinactivation data revealed striking differences in efficacy across the tested spectrum, suggesting that wavelength selection is more important than radiation dose alone. To test whether bacterial susceptibility to light varies fundamentally by spectral region, we performed a two-way ANOVA on log reduction values across fluence and wavelength variables. To contextualize these findings, *P. fungorum* photoinactivation efficacy can be compared to established data for related pathogenic *Burkholderia* species. At 254 nm, the published literature on *Burkholderia* species reveals substantial strain-dependent variation: *B. pseudomallei* strains require 1.4 mJ/cm^2^ to 7.4 mJ/cm^2^ for 4 log reduction, *B. mallei* strains 1.0 mJ/cm^2^ to 5.5 mJ/cm^2^, and *B. cepacia* strains 2.4 mJ/cm^2^ to 7.2 mJ/cm^2^, reflecting inherent differences in UVC susceptibility across the genus (Table 2). *P. fungorum*’s requirement of about 6 mJ/cm^2^ for 1 log reduction and 24.79 mJ/cm^2^ for 5.4 log reduction positions it as considerably more durable against UVC irradiation than most documented *Burkholderia* species, suggesting either enhanced DNA repair capacity, altered photoproduct formation, or structural features that reduce UV penetration [34]. This enhanced UV resistance may reflect several mechanisms. (1) There may be elevated DNA repair enzymes—particularly nucleotide excision repair (NER) systems capable of removing cyclobutane pyrimidine dimers. While there is no report of any NER systems in *P. fungorum*, numerous Gram-negative bacteria, including *Burkholderia* species, suggest it is highly likely that *P. fungorum* possesses functional NER systems. (2) Second, there may be a constitutive upregulation of stress response pathways. Notably, *P. fungorum*’s genomic architecture includes periplasmic stress response genes (*rpoE*, *rseA*, *htrA*) that may facilitate rapid cellular damage response [3,35]. This UV durability aligns with *P. fungorum*’s environmental lifestyle, where soil and aquatic habitats expose organisms to natural UV radiation, selecting for enhanced photo-resistance.

Notably, the literature documents a characteristic ‘shoulder effect’ for *B. pseudomallei*, describing an initial plateau in inactivation kinetics (until about 40 mJ/cm^2^) followed by steeper dose–response (until) about 70 mJ/cm^2^ at wavelengths in the 295 nm to 305 nm range (filtered from natural sunlight) [22]. *P. fungorum* exhibits a similar shoulder effect at 313 nm, with minimal inactivation (1.21 log reduction) at 46.8 mJ/cm^2^, followed by accelerated killing at higher fluences. This shoulder phenomenon becomes more prominent at 365 nm, where inactivation remains modest until approximately 92 J/cm^2^. This wavelength-dependent manifestation of the shoulder effect may reflect distinct photochemical mechanisms: UV radiation (222 nm, 254 nm, and 313 nm) operates primarily through direct DNA damage via cyclobutane pyrimidine dimer formation, which is a mechanism that may saturate or trigger repair responses initially [22,33].

Statistical analysis revealed the wavelength as the dominant determinant of photoinactivation efficacy with a highly significant interaction between fluence and wavelength (F = 100.4, *p* < 0.001). This finding indicates that bacterial susceptibility depends primarily on spectral selection rather than dose intensity alone. Bonferroni-corrected pairwise comparisons established a clear hierarchical ranking in efficacy across the tested spectrum—254 nm >> (222 nm ≈ 313 nm) >> 365 nm >> 400 nm >> 464 nm, with 254 nm demonstrating orders-of-magnitude superior bactericidal inactivity compared to all other wavelengths. A mechanistic analysis incorporating directly measured ROS levels revealed that ROS generation is a significantly stronger predictor of photoinactivation efficacy (F = 60.6, *p* < 0.001) than fluence dose alone (F = 24.8, *p* < 0.001). The substantial reduction in residual variance when ROS levels were incorporated into the model confirms that wavelength-dependent oxidative stress is the key factor explaining differential bacterial susceptibility across the ROS mediated spectrum of 365 nm, 400 nm, and 464 nm. These robust statistical findings (all effects *p* < 0.001 with large effect sizes surviving conservative Bonferroni correction) provide high confidence in the reliability and biological significance of the wavelength-dependent mechanisms underlying *P. fungorum* photoinactivation.

Light inactivation operates through distinctly different mechanisms, which are mediated by reactive oxygen species (ROS) generation rather than direct DNA damage. The wavelength-dependent ROS production revealed by DCFH-DA fluorescence assays directly explains the mechanistic basis of the observed efficacy hierarchy in the visible spectrum. At 365 nm, an extreme 12,642-fold fluorescence increase reflects simultaneous photon absorption by both the porphyrin UVA absorption tail [36] and flavin secondary peaks [37], generating multiple reactive oxygen species that overwhelm cellular antioxidant defenses. This extraordinary ROS production at 365 nm drives the achievement of 5.2 log reduction at substantially lower fluence (122 J/cm^2^) compared to 400 nm and 464 nm. At 400 nm, a more moderate 122-fold increase in ROS fluorescence correlates with 4.8 log reduction at 378 J/cm^2^, indicating robust but less efficient ROS generation. In contrast, 464 nm produces only a 27-fold fluorescence increase despite an applied fluence of 3017 J/cm^2^, resulting in the lowest efficacy (3.4 log reduction). The ~500-fold ROS differential (12,642 at 365 nm and 26.6 at 464 nm, without taking the fluence into account, see Figure 6) between 365 nm and 464 nm directly explains this marked inactivation efficiency gap: 365 nm generates sufficient ROS to overwhelm cellular defenses within reasonable fluence doses, whereas 464 nm ROS accumulation remains insufficient to reach bactericidal thresholds even at extended exposures. This quantitative relationship—wherein 365 nm achieves a ~47-fold greater bacterial inactivation than 464 nm and approximately a 2.7-fold greater efficacy than 400 nm—demonstrates that spectral selection is a more powerful optimization strategy than light intensity increases alone. The ROS-mediated pathway explains why efficiency declines dramatically at longer wavelengths: the spectral overlap between the light source emission and endogenous chromophore absorption spectra is the critical determinant of ROS generation rate and therefore inactivation efficacy.

The distinct wavelength-dependent ROS production reflects *P. fungorum*’s specific endogenous photosensitizer composition. At about 400 nm, the strong ROS generation (122-fold increase) correlates with the Soret band absorption (around 405 nm) of endogenous bacterial porphyrins (coproporphyrin III, protoporphyrin IX), which undergo highly efficient photon-to-ROS conversion and thereby serve as the dominant photosensitizers at this wavelength. Conversely, the reduced efficacy at 464 nm, despite the known theoretical absorption maximum of flavins around 450 nm, indicates that flavins play a subordinate role in ROS generation under the tested conditions or that the specific emission spectrum of the 464 nm LED does not optimally overlap with intracellular flavin absorption profiles. This photosensitizer selectivity—high porphyrin/low flavin—is apparently a *P. fungorum*-specific trait. The fluorescence emission spectra (Figure 7) seem to differ. The fluorescence emission spectra of riboflavin, its derivate lumichrome and NADH indicate a strong presence with their great overlap on the spectrum of *P. fungorum*. In contrast to that, no fluorescence signal of porphyrins could be detected with this method or within these conditions. However, this apparent absence of porphyrin fluorescence likely reflects spectroscopic rather than biochemical constraints: photosensitizer fluorescence is sensitive to both pH and solvent composition, and the use of different solvents for reference standards (aqueous PBS for bacterial samples versus organic solvents for porphyrin standards) introduces artifacts that can shift peak positions and alter intensities. Additionally, porphyrin conditions in aqueous solutions involve an equilibria between monomeric and aggregated forms, each exhibiting distinct fluorescence characteristics that are pH-sensitive. To definitively establish *P. fungorum*’s complete photosensitizer profile, techniques such as high-performance liquid chromatography (HPLC) coupled with mass spectrometry would provide superior chemical resolution independent of pH and solvent artifacts, thereby clarifying the quantitative and molecular basis of wavelength-dependent photoinactivation efficacy.

The DCFH-DA fluorescence assay employed in this paper provides a measurement of ROS generation with the possibility to act as a scavenger. Table 1 demonstrates that DCFH-DA might have some impact on inactivation at 365 nm, 400 nm, and 464 nm with positive inhibition values (30%, −3%, −27% respectively). Further statistical analysis including a two-way ANOVA and Bonferroni adjusted *t*-test (Table 4 and Table 7) indicate that there is no statistical factor which supports the idea of a scavenger effect. The strong correlation between measured fluorescence intensity and bacterial inactivation efficacy, particularly the ~500-fold ROS differential between 365 nm and 464 nm, indicates that DCFH-DA quantification reliably captures the relative differences in ROS production driving the observed wavelength hierarchy. The mechanistic conclusion that wavelength-dependent ROS generation is the primary driver of visible light inactivation remains robust, accounting even for singlet oxygen underestimation.

These wavelength-dependent mechanisms can be contextualized within the evolutionary ecology of *P. fungorum* and related *Burkholderia* species. *P. fungorum*’s substantial UV durability—roughly 2 to 3-fold more resistant than common B. cepacia strains—reflects its environmental lifestyle in soil and aquatic systems where natural UV radiation provides continuous selective pressure for enhanced photo-resistance. The presence of constitutive stress response genes (*rpoE*, *rseA*, *htrA*) and presumed DNA repair capacity represents an adaptation to environmental exposure rather than a consequence of antibiotic resistance mechanisms. In contrast, the limited visible light efficacy at 464 nm, despite theoretical expectations based on flavin absorption, suggests that *P. fungorum*’s photosensitizer architecture has been shaped by environmental rather than photochemical optimization. In nature, *P. fungorum* likely encounters predominantly direct UV radiation during daylight exposure with limited contribution from the longer blue wavelengths that would require robust endogenous photosensitizer systems. This ecological history explains why *P. fungorum* maintains abundant porphyrin-dependent chromoproteins or cytochrome oxidases (enabling efficient 400 nm sensitization) while apparently lacking corresponding flavin-dependent systems. Whether this photosensitizer selectivity is a constitutive, species-specific trait or an environmentally regulated response remains unknown with distinction significant implications for phototherapy protocol development.

These findings establish operational parameters for light-based *P. fungorum* decontamination across different clinical and environmental contexts. The superior efficiency of 254 nm UV (5.4 log reduction at 24.7 mJ/cm^2^) makes it optimal for rapid, energy-efficient disinfection where cost and speed are paramount. For high-throughput water treatment or surface disinfection applications, 254 nm germicidal lamps remain the gold standard. However, 365 nm offers compelling practical alternatives for long-term environmental applications: it achieves comparable log reductions (5.2 log reduction at 122.1 J/cm^2^) while generating extraordinary ROS production (12,642-fold fluorescence increase), utilizing more affordable and longer-lived UV-A lamp technology compared to far-UVC sources. From a cost–benefit perspective, 365 nm may provide superior operational value for biofilm containment in agricultural biocontrol, or water cycling systems in bioremediation applications, where replacement frequency and energy consumption are critical cost drivers. In contrast, 464 nm direct visible-light decontamination is impractical without exogenous photosensitizers: achieving equivalent log reductions to 365 nm would require over 25 times greater fluence (3017 J/cm^2^ vs. 122 J/cm^2^), rendering this approach economically unfeasible. However, hybrid visible light strategies incorporating exogenous photosensitizers (e.g., curcumin-enhanced blue light, as demonstrated for related *B. cepacia* strains achieving 4 log reduction at 28.8 J/cm^2^ [23]) could extend *P. fungorum* control to wavelengths compatible with lower-cost LED technology. For facilities where UV safety is critical (healthcare settings, food processing), a forward-looking hybrid approach combining far-UVC (222 nm, non-germicidal to human tissue) with exogenous photosensitizer augmentation at visible wavelengths might represent a promising strategy, though the 222 nm data in this study remain incomplete.

Several important limitations require discussion. First, this study employed planktonic bacterial suspensions rather than biofilm-associated *P. fungorum*, which exhibits fundamentally different physiology. Biofilms demonstrate enhanced photoresistance through matrix-mediated light scattering, reduced photon penetration into the biofilm interior, and ROS quenching by exopolysaccharide components. Whether the wavelength selectivity hierarchy (254 nm >> 222 nm ≈ 313 nm >> 365 nm >> 400 nm >> 464 nm) persists in biofilm conditions remains unknown, which represents a critical gap for environmental applications where biofilm formation is ecologically relevant. Second, the 222 nm dataset remains limited, achieving only 2.37 log reduction at maximum applied fluence, limiting mechanistic conclusions on where the typical 3 log reduction is reached. But it should be taken into account that this dataset achieved a linear phase where the missing data can be extrapolated with high confidence. Third, the study was conducted under constant temperature (8 °C during irradiation) with controlled irradiance; field applications subject to temperature fluctuations, ambient light interference, or varying bacterial physiological states may exhibit different efficacy profiles. Future research should address these limitations through the following: (1) EPR (electron paramagnetic resonance) spectroscopy for direct, real-time ROS identification independent of fluorescent probe bias; (2) HPLC-MS characterization of endogenous photosensitizer abundance and speciation in *P. fungorum* and related *Paraburkholderia* strains; (3) confocal laser scanning microscopy (CLSM) with fluorescent ROS indicators for spatiotemporal ROS visualization and intracellular localization; and (4) comparative photosensitizer profiling across environmental *Paraburkholderia* isolates to determine whether the observed porphyrin-dominant/flavin-limited profile is *P. fungorum*-specific.

## 5. Conclusions

*P. fungorum* demonstrates significant photoinactivation susceptibility across UV and visible light wavelengths with distinct mechanistic signatures at different spectral regions. At 254 nm, the bacterium exhibits robust UV resistance compared to the related literature, achieving a 5.4 log reduction at 24.79 mJ/cm^2^. Visible light inactivation operates through ROS-mediated pathways with wavelength-dependent ROS generation—validated by ANOVA and Bonferroni-corrected analysis—serving as the primary mechanistic determinant of efficacy. At 365 nm, extraordinary ROS production (12,642-fold fluorescence increase) enables the most efficient wavelength inactivation (5.2 log reduction at 122.18 J/cm^2^), while the marked inefficiency at 464 nm (3.4 log reduction at 3017 J/cm^2^) reflects limited endogenous photosensitizers (flavins or other cofactors) in the upper blue spectral region. Across all wavelengths tested, statistical analysis established a clear hierarchical ranking in inactivation efficacy: 254 nm >> (222 nm ≈ 313 nm) >> 365 nm >> 400 nm >> 464 nm with 254 nm and 365 nm representing superior inactivation wavelengths with distinct mechanisms. These findings establish *P. fungorum* as photosensitive to both UV and visible light, indicating alternatives for environmental and clinical decontamination applications.

## Figures and Tables

**Table 1:** Effect of DCFH-DA on bacterial inactivation of *P*. *fungorum* at 365 nm, 400 nm, and 464 nm. Log reduction values are presented without and with DCFH-DA along with the calculated difference (Δ log reduction), protection factor, and percentage inhibition. Positive protection factors and inhibition percentages indicate scavenging (protective) effects, while negative values indicate enhanced inactivation. Data are presented as mean values and below their respective standard error of the mean (SEM).

**365 nm**					
fluence [J/cm^2^]	0	31	61	92	122
log reduction	0	0.090	0.792	3.626	5.201
SEM ±	0	0.017	0.142	0.525	0.410
log reduction including DCFH	0	0.111	0.740	4.118	4.781
SEM ±	0	0.015	0.057	0.235	0.037
Δ log reduction	0	0.021	0.052	−0.492	0.420
protection factor		0.812	1.071	0.881	1.088
inhibition [%]		−23%	7%	−14%	8%
**400 nm**					
fluence [J/cm^2^]	0	126	252	378	
log reduction	0	1.275	3.973	5.909	
SEM ±	0	0.319	0.666	0.713	
log reduction including DCFH	0	0.759	3.389	4.854	
SEM ±	0	0.238	0.177	0.597	
Δ log reduction	0	0.517	0.584	1.055	
protection factor		1.681	1.172	1.217	
inhibition [%]		41%	15%	18%	
**464 nm**					
fluence [J/cm^2^]	0	1006	2012	3017	
log reduction	0	0.848	2.159	3.416	
SEM ±	0	0.205	0.334	0.242	
log reduction including DCFH	0	0.590	2.214	4.332	
SEM ±	0	0.268	0.576	0.550	
Δ log reduction	0	0.258	−0.056	−0.916	
protection factor		1.438	0.975	0.789	
inhibition [%]		30%	−3%	−27%	

**Table 2:** Summary of UV and visible light photoinactivation data for the close relative Burkholderia species from the literature. Reduction values (presented as both logarithmic reduction and percentage bacterial inactivation), mechanisms of inhibition, and corresponding fluence doses are presented. Data for *B. cepacia*, *B. pseudomallei*, and *B. mallei* were compiled from published UV disinfection and photodynamic inactivation studies. The mechanism column distinguishes between direct DNA damage and ROS-mediated inactivation. A ROS-mediated approach enhanced with exogenous photosensitizers is listed, too. The “comment” column specifies the data source format, and sources are cited accordingly.

*Burkholder* Strains	Irradiation Wavelength	Reduction in [log] and [%]	Mechanism of Inhibition	Fluence	Comment	Source
*B. cepacia* R-1464	254 nm	3.8 log, 99.98%	DNA damage	2.4 mJ/cm^2^	from diagram	[21]
*B. cepacia* LMG 13010	254 nm	2.5 log, 99.68%	DNA damage	7.2 mJ/cm^2^		
*B. cepacia* LMG 12615	254 nm	1.5 log, 96.84%		7.2 mJ/cm^2^		
*B. cepacia* LMG 14291	254 nm	1.4 log, 96.02%	DNA damage	7.2 mJ/cm^2^		
*B. cepacia* LMG 16232	254 nm	2 log, 99%	DNA damage	7.2 mJ/cm^2^		
*B. cepacia* ATCC.25416	425 nm	4 log, 99.99%	ROS-mediated and enhanced with exogenous photosensitizers: 50 µM curcumin + 0.4% EDTA	28.8 J/cm^2^	from text	[23]
*B. pseudomallei* NCTC 13177	295 nm to 305 nm	per 1 log, 90%		26.5 mJ/cm^2^	from text, mentions shoulder effect	[22]
*B. pseudomallei* NCTC 13177	254 nm	1 log, 90%	DNA damage	4.4 mJ/cm^2^	table format	[32,33]
		4 log, 99.99%	ROS mediated	13 mJ/cm^2^		
*B. Mallei* M-9	254 nm	1 log, 90%	DNA damage	1 mJ/cm^2^	table format	[34]
		2 log, 99%		2.4 mJ/cm^2^		
		3 log, 99.9%		3.8 mJ/cm^2^		
		4 log, 99.99%		5.2 mJ/cm^2^		
*B. mallei* M-13	254 nm	1 log, 90%		1.2 mJ/cm^2^		
		2 log, 99%		2.7 mJ/cm^2^		
		3 log, 99.9%		4.1 mJ/cm^2^		
		4 log, 99.99%		5.5 mJ/cm^2^		
*B. pseudomallei* CA650	254 nm	1 log, 90%		1.4 mJ/cm^2^		
		2 log, 99%		2.8 mJ/cm^2^		
		3 log, 99.9%		4.3 mJ/cm^2^		
		4 log, 99.99%		5.7 mJ/cm^2^		
*B. pseudomallei* ATCC 11688	254 nm	1 log, 90%		1.7 mJ/cm^2^		
		2 log, 99%		3.5 mJ/cm^2^		
		3 log, 99.9%		5.5 mJ/cm^2^		
		4 log, 99.99%		7.4 mJ/cm^2^		

**Table 3:** Two-way ANOVA analysis of fluence and wavelength effects on *P. fungorum* log reduction. Df, degrees of freedom; Sum Sq, sum of squares (total variance attributed to each source); Mean Sq, mean square (variance per degree of freedom = Sum Sq/Df); F-value, F-statistic (ratio of factor Mean Sq to Residual Mean Sq). All main effects and interaction were highly significant (*p* < 0.001, ***), demonstrating that both fluence and wavelength independently influence bacterial log reduction with a wavelength-dependent dose–response relationship. The dominant interaction term (F = 100.4, Sum Sq = 416.2) demonstrates wavelength-dependent dose–response relationships, reflecting mechanistically distinct photochemical pathways (UV-mediated direct DNA damage vs. ROS-mediated visible light mechanisms). Variance partitioning (η^2^): wavelength = 17.3%, fluence = 4.2%, interaction = 61.3%, revealing that spectral selectivity dominates over light intensity effects (wavelength × fluence interaction is 14.6× more influential than fluence alone). Residual variance (116.9, ~21% of total) attributable to biological heterogeneity and experimental noise; the model explains 79% of the observed log reduction variation. Sample size: n = 147 (six observations with missing values excluded from original n = 153).

	Df	Sum Sq	Mean Sq	F-Value	*p*-Value
fluence [mJ/cm^2^]	1	28.2	28.2	34	3.49 × 10^−8^ ***
wavelength	5	117.2	23.4	28.2	<2 × 10^−16^ ***
fluence × wavelength (interaction)	5	416.2	83.2	100.4	<2 × 10^−16^ ***
residuals	141	116.9	0.83		

**Table 4:** Two-way ANOVA analysis of fluence, wavelength, and DCFH-DA treatment effect on *P. fungorum* log reduction (only 365 nm, 400 nm, 464 nm; n = 117). Df, degrees of freedom; Sum Sq, sum of squares; Mean Sq, mean square; F-value, F-statistic; *** *p* < 0.001; ns, not significant (*p* > 0.05). DCFH-DA treatment status shows no significant effect (F = 0.374, *p* = 0.542), validating the fluorescent ROS probe as non-interfering with antimicrobial efficacy. Fluence and wavelength remain highly significant (*p* < 0.001), while the wavelength × fluence interaction dominates (F = 155.7, Sum Sq = 302.0), demonstrating that spectral selectivity persists as the primary efficacy determinant within the visible spectrum.

	Df	Sum Sq	Mean Sq	F-Value	*p*-Value
fluence [mJ/cm^2^]	1	28.5	28.5	29.3	3.54 × 10^−7^ ***
wavelength	2	97.4	48.7	50.2	3.20 × 10^−16^ ***
DCFH-DA added	1	0.36	0.36	0.37	0.542 (ns)
fluence × wavelength (interaction)	2	302	151	155.7	<2 × 10^−16^ ***
residuals	110	106.6	0.97		

**Table 5:** Two-way ANOVA with ROS level as continuous covariate: analysis of fluence and wavelength effects on *P. fungorum* log reduction within visible wavelengths (365, 400, 464 nm; n = 35 observations with complete ROS and CFU data). Df, degrees of freedom; Sum Sq, sum of squares; Mean Sq, mean square; F-value, F-statistic; *** *p* < 0.001. ROS level emerges as the dominant predictor (F = 60.60, *** *p* = 1.76 × 10^−8^, Mean Sq = 37.3), exceeding fluence (Mean Sq = 15.3) by 2.4-fold, demonstrating that ROS generation is a stronger mechanistic driver than light dose. All main effects and interaction remain highly significant, indicating partially mediated relationships: ROS is the primary mechanistic intermediate, but wavelength also exerts independent effects through wavelength-specific photochemical processes. Residual variance decreased dramatically (from 106.6 to 17.2 units; 6.2-fold reduction) upon ROS incorporation, indicating that measured ROS levels explain an additional 89.4 units (η^2^ = 0.839, representing 83.9% of previously unexplained variance), establishing ROS as the quantitative basis of wavelength-dependent photoinactivation heterogeneity.

	Df	Sum Sq	Mean Sq	F-Value	*p*-Value
fluence [mJ/cm^2^]	1	15.3	15.3	24.8	2.90 × 10^−5^ ***
wavelength	2	31.4	15.7	25.5	4.85 × 10^−7^ ***
ROS level	1	37.3	37.3	60.6	1.76 × 10^−8^ ***
fluence × wavelength (interaction)	2	47.3	23.5	38.2	9.94 × 10^−9^ ***
residuals	28	17.2	0.62		

**Table 6:** Estimated marginal means and 95% confidence intervals for log reduction by wavelength (all six wavelengths: 222–464 nm), Bonferroni corrected for 15 pairwise contrasts (adjusted α = 0.00333). Emmean, estimated marginal mean at mean fluence level; SE, standard error; CI, confidence interval. A clear wavelength hierarchy is evident: 254 nm >> 313 nm ≈ 222 nm >> 365 nm >> 400 nm >> 464 nm. The 254 nm wavelength (emmean = −86,905) significantly exceeds all others (all *p* < 0.0001); 313 nm and 222 nm show equivalent efficacy (*p* = 1.000), indicating similar damage produced with different mechanisms; visible wavelengths show progressive efficacy decline with increasing wavelength. All comparisons reached stringent significance, confirming true spectral differences rather than statistical artifacts.

Wavelength [nm]	emmean (±SE)	Df	Lower CI	Upper CI
222	−28,935 (±8430)	141	−45,600	−12,263
254	−86,905 (±11,100)	141	−109,000	−64,871
313	−16,204 (±1970)	141	−20,100	−12,314
365	−15 (±1.1)	141	−17.8	−13.1
400	−5 (±0.2)	141	−6.2	−5.3
464	−0.3 (±0.2)	141	−0.7	0.1

**Table 7:** Estimated marginal means and 95% confidence intervals for log reduction by the wavelengths 365 nm, 400 nm, and 464 nm, averaged over DCFH-DA treatment status (presence/absence), Bonferroni-corrected for 3 pairwise contrasts (adjusted α = 0.0167). Emmean, estimated marginal means; SE, standard error; CI, confidence interval. All three comparisons remain highly significant (*p* < 0.0001): 365 nm (emmean = −20) significantly exceeds both 400 nm (3-fold difference, *p* < 0.0001) and 464 nm (50-fold difference, *p* < 0.0001); 400 nm exceeds 464 nm (17-fold difference, *p* < 0.0001). Consistency with Table 6 visible-light rankings confirms the robustness of wavelength efficacy hierarchy independent of analytical stratification.

Wavelength [nm]	emmean (±SE)	Df	Lower CI	Upper CI
365	−20 (±1.7)	110	−24	−17
400	−7.6 (±0.3)	110	−8.3	−6.9
464	−0.4 (±0.2)	110	−0.8	0.03

**Table 8:** Estimated marginal means and 95% confidence intervals for log reduction by wavelength within the spectrum of 365 nm, 400 nm, and 464 nm, DCFH-positive samples with complete ROS data; n = 35), adjusted for ROS level as continuous covariate, Bonferroni corrected for 3 pairwise contrasts (adjusted α = 0.0167). Emmean, estimated marginal means adjusted for ROS level; SE, standard error; CI, confidence interval. All three comparisons remain significant (*p* ≤ 0.0027) after ROS adjustment. Notably, the 365 nm versus 400 nm difference increased by approximately 60% after adjustment for ROS (−19.3 versus −12.3 units; compare Table 7), indicating that 365 nm generates disproportionately high ROS relative to 400 nm. This amplification reveals that wavelength-specific ROS production—not wavelength per se—drives the observed efficacy hierarchy, establishing ROS as the quantitative mechanistic basis of visible-light photoinactivation selectivity.

Wavelength [nm]	emmean (±SE)	Df	Lower CI	Upper CI
365	−27(±5.1)	28	−37	−16
400	−7.7 (±0.7)	28	−9.1	−6.2
464	−0.4 (±0.3)	28	−1	0.2

## Data Availability

The data are available on reasonable request.
